# Circular RNA circ_0020710 drives tumor progression and immune evasion by regulating the miR-370-3p/CXCL12 axis in melanoma

**DOI:** 10.1186/s12943-020-01191-9

**Published:** 2020-05-07

**Authors:** Chuan-Yuan Wei, Meng-Xuan Zhu, Nan-Hang Lu, Jia-Qi Liu, Yan-Wen Yang, Yong Zhang, Yue-Dong Shi, Zi-Hao Feng, Jia-Xia Li, Fa-Zhi Qi, Jian-Ying Gu

**Affiliations:** 1 Department of Plastic Surgery, Zhongshan Hospital, Fudan University, Shanghai, 200032 P. R. China; 2 Department of Medical Oncology, Zhongshan Hospital, Fudan University, Shanghai, 200032 P. R. China; 3 Department of Neurology, Hefei Affiliated Hospital of Anhui Medical University, Hefei, Anhui 230000 P. R. China

**Keywords:** circRNAs, CXCL12, Immune suppression, Melanoma

## Abstract

**Background:**

Circular RNAs (circRNAs) have been reported to have critical regulatory roles in tumor biology. However, their contribution to melanoma remains largely unknown.

**Methods:**

CircRNAs derived from oncogene CD151 were detected and verified by analyzing a large number of melanoma samples through quantitative real-time polymerase chain reaction (qRT-PCR). Melanoma cells were stably transfected with lentiviruses using circ_0020710 interference or overexpression plasmid, and then CCK-8, colony formation, wound healing, transwell invasion assays, and mouse xenograft models were employed to assess the potential role of circ_0020710. RNA immunoprecipitation, luciferase reporter assay and fluorescence in situ hybridization were used to evaluate the underlying mechanism of circ_0020710.

**Results:**

Our findings indicated that circ_0020710 was generally overexpressed in melanoma tissues, and high level of circ_0020710 was positively correlated with malignant phenotype and poor prognosis of melanoma patients. Elevated circ_0020710 promoted melanoma cell proliferation, migration and invasion in vitro as well as tumor growth in vivo. Mechanistically, we found that high level of circ_0020710 could upregulate the CXCL12 expression via sponging miR-370-3p. CXCL12 downregulation could reverse the malignant behavior of melanoma cells conferred by circ_0020710 over expression. Moreover, we also found that elevated circ_0020710 was correlated with cytotoxic lymphocyte exhaustion, and a combination of AMD3100 (the CXCL12/CXCR4 axis inhibitor) and anti-PD-1 significantly attenuated tumor growth.

**Conclusions:**

Elevated circ_0020710 drives tumor progression via the miR-370-3p/CXCL12 axis, and circ_0020710 is a potential target for melanoma treatment.

## Background

Over the past years, the prognosis of melanoma patients has improved largely. Unfortunately, only a few patients are able to achieve a sustained response, and most will eventually succumb to these diseases, with a 5-year overall survival (OS) rate of 30%~ 40% [1]. According to the statistics from the American Cancer Society, melanoma is still the most lethal type of skin tumor with an estimated 91,270 new cases and 9320 deaths in 2018 [2]. Therefore, further research is urgently needed to identify more effective therapeutic schedules in order to achieve better clinical outcomes in melanoma.

CircRNAs are endogenous non-coding RNAs (ncRNAs). Unlike linear RNA terminating with a 5’cap and a 3′ poly (A) tail, circRNAs have a covalently closed-loop structure [3]. Due to this property, circRNAs have strong resistance to exonucleolytic degradation and maintain high cellular stability [4, 5]. However, circRNAs were considered to be the result of the back-splicing or by-product of pre-mRNA processing with low abundance over the past years [6]. With the development of advanced RNA sequencing (RNA-seq) technology and the improvement of the algorithm for circRNA detection, circRNAs have recently been pushed to the spotlight, and they are tightly linked to many physiological and pathological processes, including tumorigenesis, tumor development and immune escape [7]. For example, circTRIM33–12 is significantly downregulated in hepatocellular carcinoma tissues, and circTRIM33–12 upregulates TET1 expression by sponging miR-191, resulting in significantly reduced 5-hydroxymethylcytosine levels [8]. However, few studies have linked circRNAs to the biological behavior of melanoma, which is of great significance.

CD151, a member of the transmembrane-4 family, encodes a protein whose open reading frame contains 253 amino acids. CD151 is mainly concentrated on the cell membrane and expressed in almost all cell types and tissues [9]. As a critical transmembrane protein (such as integrins and growth factor receptors) activity organizer and regulator, CD151 is tightly linked to many physiological and pathological processes, especially in oncogenesis and cancer development [10]. For example, previous studies have shown that elevated CD151 induces the epithelial-mesenchymal transition (EMT) and neoangiogenesis of hepatocellular carcinoma via amplifying integrin α6β1 to PI3K pathway [11, 12]. In melanoma, it has been demonstrated that CD151 affinity interaction stimulates integrin-dependent signal transduction, resulting in increased cell migration and MMP-9 expression [13]. Now, overexpression of CD151 has been found in almost all types of tumors [14]. Thus, CD151, as an oncoprotein, plays an important role in tumor progression, including melanoma.

In this study, we identified a novel circRNA derived from the CD151, termed circ_0020710, was significantly overexpressed in melanoma tissues compared with matched normal tissues and benign nevi tissues, and high circ_0020710 level was positively correlated with poor prognosis of melanoma patients. Mechanistically, elevated circ_0020710 level upregulated CXCL12 expression to promote melanoma progression and induce immune evasion by sponging miR-370-3p. Our findings suggest that circ_0020710 functions as a promoter in melanoma progression and maybe a potential target in melanoma treatment.

## Methods

### Patients and samples

A total of 88 melanoma and matched normal tissues and 18 benign nevi tissues were randomly gathered from the Department of Plastic & Reconstructive Surgery of Zhongshan Hospital, Fudan University (Shanghai, China). All samples were frozen in liquid nitrogen and then stored stably at − 80 °C. All patients underwent total resection and were verified histologically and pathologically by two pathologists. Before the operation, no one had received any form of radiotherapy or chemotherapy, and all patients obtained detailed clinicopathological and follow-up data. This study was approved by the Ethics Committee of Zhongshan Hospital, Fudan University, and each patient provided written informed consent.

### Cell culture and transfection

A375, A2058, A875, Sk-mel-28, MV3, M14 (melanoma cell lines), HaCaT (normal epidermal cell line) and HEK-293 T cells were purchased from the Cell Bank of the Chinese Academy of Sciences (Shanghai, China), incubated at 37 °C with 5% CO_2_ and determined to be mycoplasma-free. Small hairpin RNAs (shRNAs) and complementary DNA (cDNA) plasmids of circ_0020710, and small interfering RNA (siRNA) of CXCL12 were purchased from Genomeditech Co., Ltd. (Shanghai, China). The transfected cells were screened with puromycin (2 μg/ml) for 1 week to establish stable cell lines. The miR-370-3p mimics and control plasmids were purchased from GeneChem Co., Ltd. (Shanghai, China), and transiently transfected with lipofectamine 2000 reagent (Invitrogen, Carlsbad, CA, USA) according to the manufacturer’s instructions. The target sequences are shown in Table S3.

### QRT-PCR and western blot assays

QRT-PCR and western blot analyses were performed as our previous study and the protocol is given in the Supplementary Materials and Methods [15]. The primers and antibodies are presented in Table S1 and S2.

### Immunohistochemistry (IHC) and fluorescence in situ hybridization (FISH)

IHC assay was performed as described in our previous study and the protocol is given in the Supplementary Materials and Methods [16]. The antibodies used in this study are presented in Table S2. For the FISH assay, circ_0020710 and miR-370-3p were captured with Alexa 488-labeled and Cy3-labeled probes (Genepharma Co., Ltd. Shanghai, China), respectively. After prehybridization, circ_0020710 and miR-370-3p probes were hybridized in the prepared hybridization buffer, and the nuclei were stained with DAPI (Yeasen, Shanghai, China), and circ_0020710 and miR-370-3p were detected using a confocal microscope.

### CCK-8, colony formation, wound healing migration, and transwell invasion assays

CCK-8, colony formation, wound healing migration, and transwell invasion assays were performed as described in our previous studies and the protocols are given in Supplementary Materials and Methods [17, 18].

### CircRNAs in vivo precipitation (circRIP)

CircRIP assay was performed using a Magna RIP RNA-Binding Protein Immunoprecipitation Kit (Millipore, Billerica, MA, USA) as described in our previous study [8]. Biotin-labeled circ_0020710 (Tables S4) was synthesized by Sangon Biotech (Shanghai, China). Briefly, circ_0020710-overexpressing cells were washed with PBS, fixed in 1% formaldehyde, lysed in Co-IP buffer, sonicated and finally centrifuged. The supernatant (50 μL) was used as input and the rest was incubated with a probes-streptavidin-dynabeads (M-280; Invitrogen, Carlsbad, CA, USA) mixture at 30 °C for 12 h. The mixture was incubated with lysis buffer and proteinase K. Then the RNA was extracted using TRIzol Reagent (Invitrogen, Carlsbad, CA, USA) and used for further studies.

### Luciferase reporter assay

The vectors of luciferase reporters were synthesized using a Mutagenesis Kit (QIAGEN, California, USA). Briefly, cells were inoculated into a 96-well plate and were co-transfected with miR-370-3p mimics or the negative control and the luciferase reporter vector as well as lipofectamine 2000 reagent (Invitrogen). After 48 h, relative luciferase activity was normalized to Renilla luciferase activity.

### In vivo assays

All of the animal experiments were approved by the Animal Experimentation Ethics Committee of Zhongshan Hospital, Fudan University. Six-week-old C57BL/6 mice were maintained according to the guidelines of the 3Rs (replacement, reduction, and refinement). A total of 5 × 10^6^ cells resuspended in 100 μL of PBS were inoculated subcutaneously in the left flank of the mice. After the tumor was detected, tumor size was measured every 3 days by a vernier caliper and tumor volume was calculated as volume (cm^3^) = L x W^2^ × 0.5 with L and W representing the largest and smallest diameters, respectively. Animals were euthanized when the tumor volumes reached a maximum of 2000 mm^3^.

### Statistical analysis

Statistical analyses were performed using the SPSS software version 19.0 (SPSS, Inc., Chicago, IL) or GraphPad Prism 7.0 (GraphPad Software, USA) as previously described [16]. The values are presented as the mean ± standard deviation (SD). For comparisons, the chi-squared test, student’s t-test, Mann-Whitney U test, Kruskal-Wallis test and one-way ANOVA test were performed, as appropriate. The survival curve was prepared using the Kaplan-Meier method and analyzed by the log-rank t-test. Cox’s proportional hazard regression model was used to analyze the independent prognostic factors. *P* < 0.05 was considered statistically significant.

## Results

### Analysis of CD151-derived circRNAs in melanoma

**Fig. 1:**
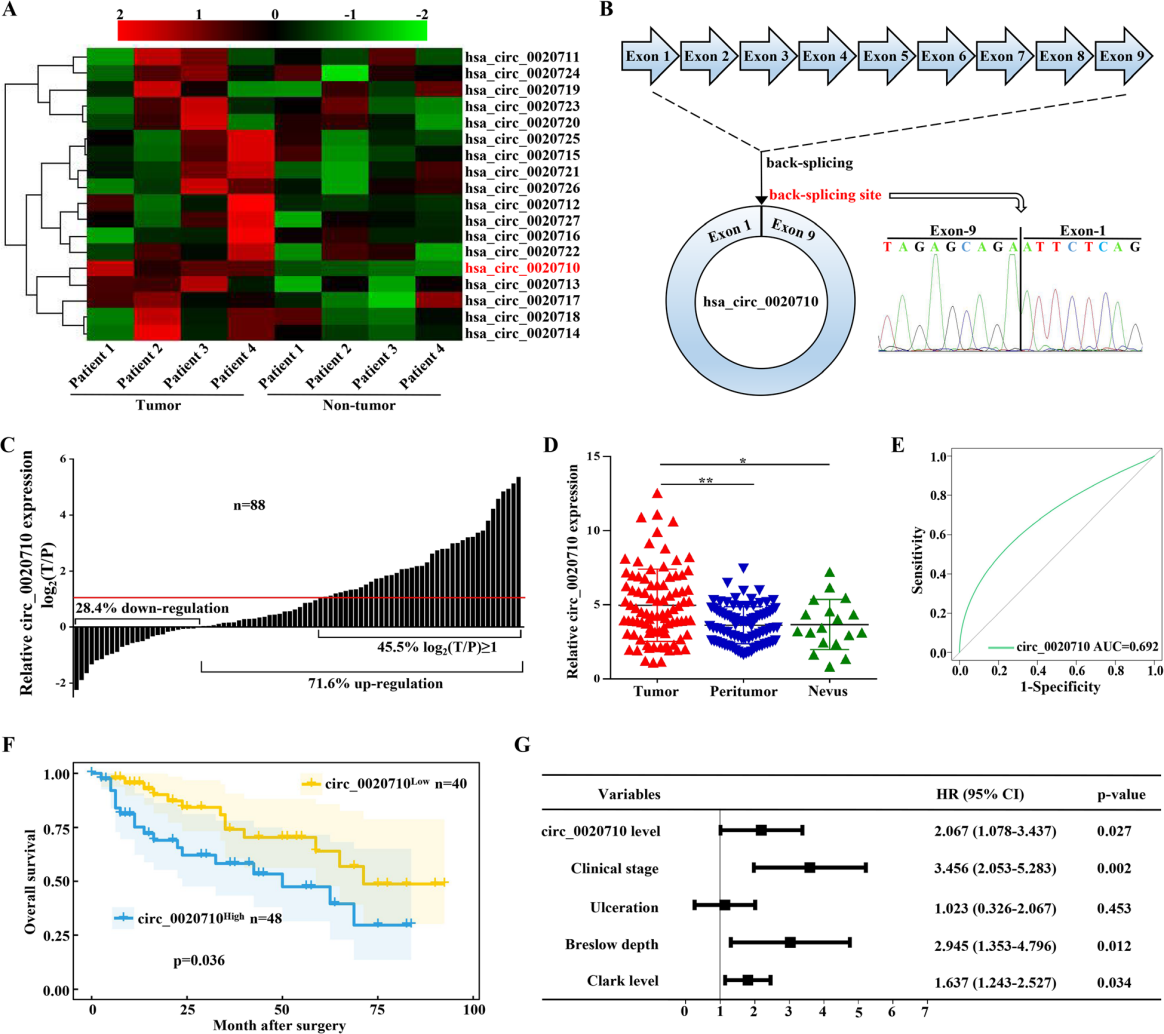
Analyses of the CD151-derived circRNA expression and prognosis in melanoma. **a** The heatmap shows the CD151-derived circRNA expression of four melanoma and non-tumor tissues analyzed by qRT-PCR. **b** Schematic illustration demonstrates circ_0020710 formation via exons 1–9 circularization of the CD151 gene. The presence of circ_0020710 was validated by qRT-PCR, followed by Sanger sequencing. **c** and **d** The relative expression of circ_0020710 in 88 paired melanoma and normal tissues and 18 benign nevi tissues was analyzed by qRT-PCR. Paired student’s t-test and Mann-Whitney U test were used for the statistical analyses. **e** The AUC of the ROC curve in distinguishing melanoma and normal tissues. **f** Based on the expression of circ_0020710, the overall survival curve was performed using Kaplan-Meier methods and analyzed by the log-rank test. **g** Multivariate analyses of factors associated with OS. *p < 0.05; **p < 0.01

Previous studies have shown that CD151 is overexpressed in multiple tumors and participates in tumor progression [9, 11–13]. Herein, we detected the level of CD151-derived circRNAs from four paired human melanoma samples, and results showed that circ_0020710 was the most significantly overexpressed circRNAs in melanoma tissues compared with matched normal tissues (Fig. 1a). By comparing the RNA sequence of circ_0020710 and CD151 from circBase, we found that circ_0020710 is looped and comprised of exons 1–9 of CD151 gene with a length of 1545 bp (on chr11: 832,951-838,831). We confirmed the head-to-tail splicing of circ_0020710 via Sanger sequencing (Fig. 1b). Moreover, we found that circ_0020710 was more resistant to RNase R than linear CD151 in two randomly selected melanoma cell lines (Fig. S1a, b).

We further explored the clinical significance of circ_0020710 in melanoma samples. Among the 88 paired tissues, we found that circ_0020710 expression was higher in 63 (71.6%), and significantly higher (log_2_FC≧1) in 40 (45.5%) melanoma tissues than that in paired normal tissues (Fig. 1c and d). Additionally, we showed that circ_0020710 expression was upregulated in melanoma tissues compared with benign nevi tissues (Fig. 1d). Then, we prepared the receiver operating characteristic (ROC) curve and found that the area under the curve (AUC) of circ_0020710 in distinguishing melanoma samples and normal ones was 0.692, indicating a good prediction model (Fig. 1e). Furthermore, we investigated the relationship between circ_0020710 expression and clinical characteristics in 88 melanoma patients, indicating that forced circ_0020710 expression was significantly correlated with the advanced Breslow depth (*p* = 0.028) and Clark level (*p* = 0.035, Table 1). Moreover, patients with high circ_0020710 expression (*n* = 48) had shorter OS than those with low circ_0020710 expression (*n* = 40) (*p* = 0.036, Fig. 1f). Importantly, Cox regression analysis indicated that the clinical stage, Breslow depth, Clark level, and the elevated circ_0020710 level are independent prognostic factors for melanoma patients (Fig. 1g and Table 2). Together, these results suggest that circ_0020710 could be a critical promoter of melanoma progression.

**Table 1:** Correlations Between circCD151 Expression and Clinicopathologic Features in 88 Melanoma Patients

Variable	Number of Patients		*p*-value*
	circCD151^low^	circCD151^high^	
Age, year			
<60	16	20	0.874
≥60	24	28	
Gender			
Male	24	27	0.723
Female	16	21	
Anatomic site			
Acra	29	37	0.388
Trunk	4	7	
Other	7	4	
Ulceration			
Present	6	10	0.480
Absent	34	38	
Breslow depth(mm)			
≤2	19	12	0.028
>2	21	36	
Clark level			
I-III	24	18	0.035
IV-V	16	30	
Lymph nodes metastasis			
No	30	32	0.394
Yes	10	16	
Distant metastasis			
No	35	40	0.221
Yes	5	8	
Clinical stage			
I-II	34	33	0.075
III-IV	6	15	

A chi-square test was used for comparing groups between low and high circCD151 expression

* *p* < 0.05 was regarded as statistically significant

**Table 2:** Univariate and Multivariate Analyses of Factors Associated With OS

Variable	Univariate		Multivariate Analysis	
	P*	HR	95%CI	P*
Age, year (≥60 vs. <60)	0.395			NA
Gender (Men vs. Women)	0.623			NA
Anatomic site (Acra vs. Trunk vs. Other)	0.723			NA
Ulceration (Present vs. Absent)	0.045			NS
Breslow depth(mm) (>2 vs. ≤2)	0.019	2.945	1.353-4.796	0.012
Clark level (IV-V vs. I-III )	0.028	1.637	1.243-2.527	0.034
Lymph nodes metastasis (Yes vs. No )	0.247			NA
Distant metastasis (Yes vs. No)	0.106			NA
Clinical stage (III-IV vs. I-II)	<0.001	3.456	2.053-5.283	0.002
circCD151 staining (High vs. Low)	0.015	2.067	1.078-3.437	0.027

*OS* overall survival, *NS* not significant, *NA* not adopt

* *p* < 0.05 was regarded as statistically significant, the *p*-value was calculated using Cox proportional hazards regression

### Circ_0020710 promotes the proliferation, migration and invasion of melanoma cells

**Fig. 2:**
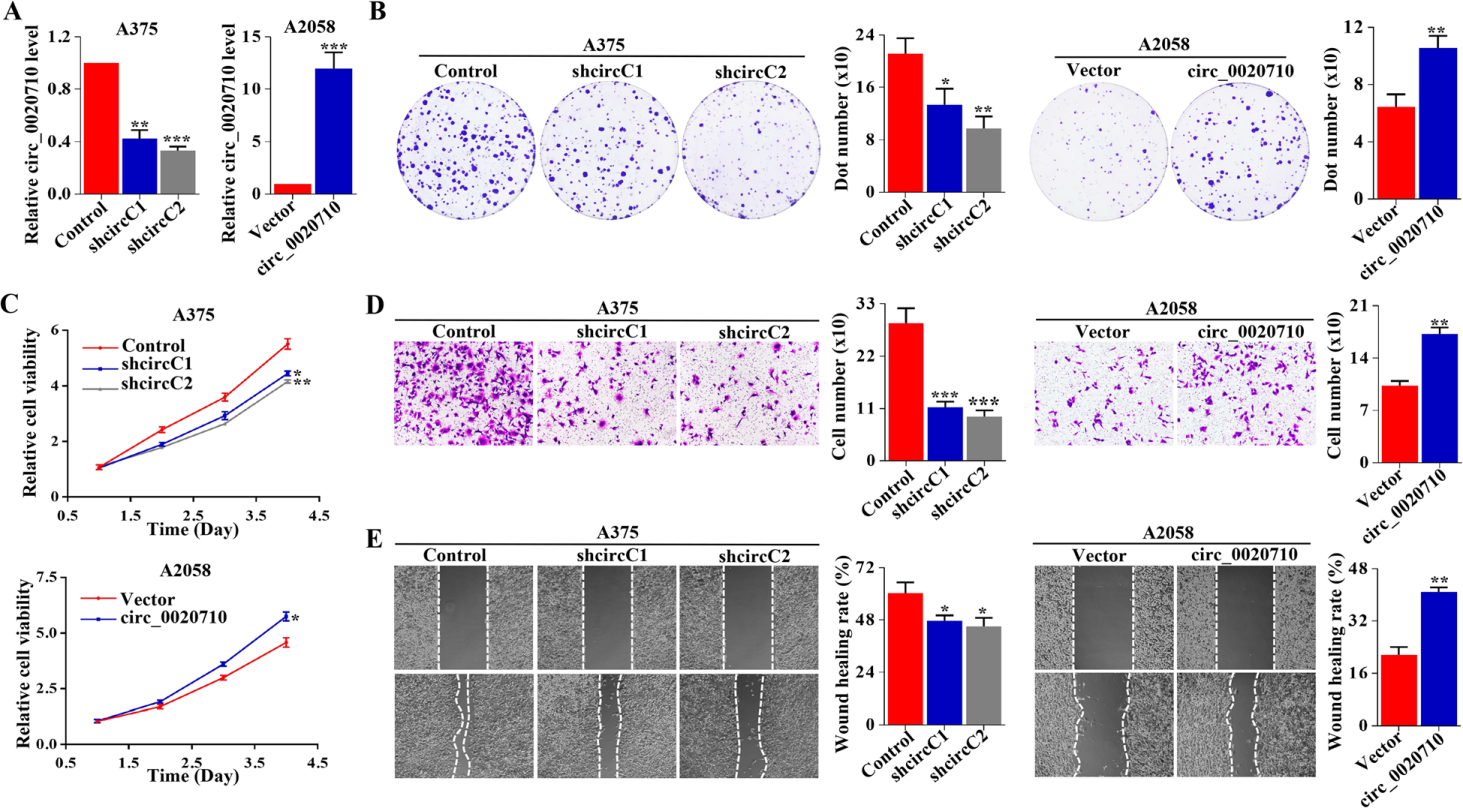
Elevated circ_0020710 promotes melanoma progression. **a** The efficacy of circ_0020710 interference and overexpression was analyzed by qRT-PCR. **b** Colony formation assay was used to detect the proliferation ability of melanoma cells with different treatments. **c** CCK-8 assay was performed to detect the proliferation of melanoma cells. **d** Transwell invasion assay was used to detect the invasion ability of melanoma cells following different treatments. **e** Wound healing migration assay was performed to detect the migration ability of melanoma cells with different treatments. Unpaired student’s t-test, Mann-Whitney U test, Kruskal-Wallis test and one-way ANOVA test were used for the statistical analyses. *p < 0.05; **p < 0.01; ***p < 0.001

To explore the biological function of circ_0020710, we conducted a series of in vitro experiments. We detected circ_0020710 expression by qRT-PCR, and found that circ_0020710 level was generally higher in melanoma cell lines compared with that in HaCaT, a normal epidermal cell line (Fig. S2a). We designed two shRNAs specifically targeting the circ_0020710 back-splice junction site (designated shcircC1–2). Compared with the control shRNA (designated Control), circ_0020710 expression was significantly down-regulated by circ_0020710 shRNAs in A375 cell lines (with the highest endogenous circ_0020710 level) (Fig. 2a). Using the plasmid vector, we succeeded in over-expressing circ_0020710 level in A2058 cells (with the lowest endogenous circ_0020710 level). However, the CD151 mRNA level was not influenced by circ_0020710 expression (Fig. S2b). CCK-8 and colony formation assays showed that the cell viability was inhibited after circ_0020710 downregulation, while reversed by circ_0020710 overexpression (Fig. 2b and c). Wound-healing migration and transwell invasion assays revealed that circ_0020710 knockdown decreased, while circ_0020710 overexpression increased melanoma cell migration and invasion (Fig. 2d and e). Additionally, we performed western blot assays and showed that elevated circ_0020710 increased the level of PCNA, CDK2, while without affecting the expression of CDK1 (Fig. S2c). Taken together, these results show that elevated circ_0020710 level promotes melanoma progression in vitro.

### circ_0020710 acted as a miR-370-3p sponge in melanoma cells

**Fig. 3:**
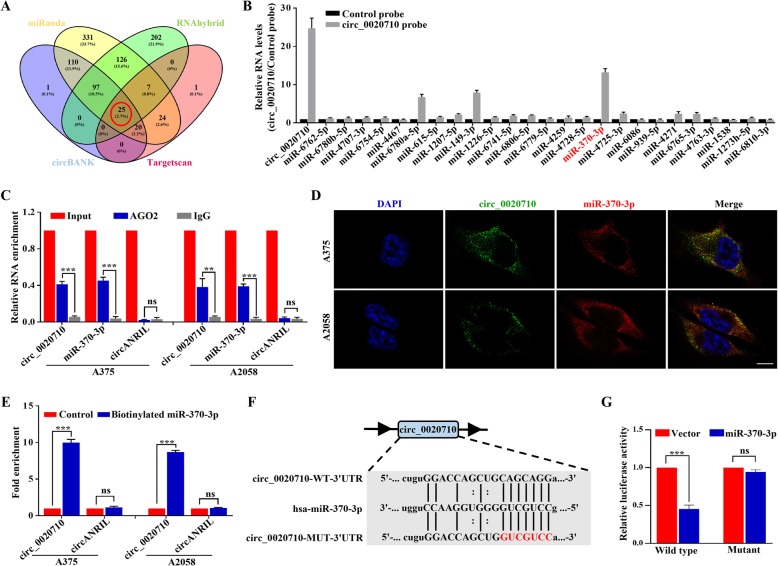
circ_0020710 acts as a sponge of miR-370-3p. **a** Schematic illustration exhibiting the overlap of circ_0020710 target miRNAs, predicted by miRanda, circbank, TargetScan, and RNAhybrid databases. **b** CircRIP was performed in A2058-circ_0020710 cells using circ_0020710 and negative control probes. **c** RIP experiments were carried out using an AGO2 antibody. **d** circ_0020710 and miR-370-3p were detected by FISH assay. **e** circ_0020710 level in the streptavidin-captured fractions from the melanoma cell lysates after transfection with biotinylated miR-370-3p or negative control. **f** Putative binding site of miR-370-3p with respect to circ_0020710 was predicated via StarBase v3.0. **g** The luciferase activity of pLG3-circ_0020710 in HEK-293 T cells after co-transfection with miR-370-3p. Unpaired student’s t-test and Mann-Whitney U test were used for the statistical analyses. **p < 0.01; ***p < 0.001; ns, no significant

Increasing studies have shown that circRNAs participated in tumor progression mainly through their function of miRNA sponging [19]. Therefore, we speculated that circ_0020710 could sponge to certain miRNAs that might play certain roles in melanoma development. Through the Nuclear/Cytosol Fractionation assay, we demonstrated that circ_0020710 was mainly localized in the cytoplasm of melanoma cells (Fig. S3a and b). We then carried out the RNA immunoprecipitation assay (RIP) with an argonaute 2 (AGO2) antibody in HEK-293 T cells. The result showed that circ_0020710, not circANRIL (a circRNA does not bind to AGO2), was significantly enriched by the AGO2 antibody (Fig. S3c), suggesting that circ_0020710 binds and interacts with miRNAs. Four databases (including miRanda, circBank, TargetScan, and RNAhybrid) were then employed to predict the potential targets of circ_0020710, and 25 miRNAs were overlapped in this four databases (Fig. 3a). To confirm the interaction between circ_0020710 and miRNAs, a circRNA-specific probe was designed to perform circRIP assay in A2058-circ_0020710 cells. The result showed that circ_0020710 and miR-370-3p were obviously enriched, whereas other miRNAs were not, indicating that miR-370-3p was one of the critical circ_0020710-associated miRNAs in melanoma cells (Fig. 3b). Next, we carried out a RIP assay with the AGO2 antibody in A375 and A2058 cells. Our results showed that circ_0020710 and miR-370-3p, but not circANRIL, were significantly enriched by the AGO2 antibody (Fig. 3c). These results indicated that circ_0020710 might act as a binding platform for miR-370-3p. By FISH analysis, we further confirmed that both circ_0020710 and miR-370-3p were mainly located in the cytoplasm of A2058 and A375 cells, and importantly, they were obviously co-localized (Fig. 3d). To further verify the interaction between miR-370-3p and circ_0020710, we designed a specific biotinylated miR-370-3p probe to perform RIP assay. The results showed that the biotinylated miR-370-3p probe could effectively capture circ_0020710 (Fig. 3e). Moreover, we carried out luciferase reporter assays via transfecting HEK-293 T and A2058 cells with luciferase reporter vectors (containing the wild type or mutant sequence of miR-370-3p target). Compared with that of the mutant sequence, the luciferase reporter activity was significantly decreased by the miR-370-3p mimics in cells transfected with the wild type sequence (Fig. 3f and g, Fig. S3d). Meanwhile, after overexpressing or silencing circ_0020710, the level of miR-370-3p did not change, and the level of circ_0020710 also remained unchanged after miR-381-3p was up regulated or downregulated (Fig. S3e and f). Together, our results provide evidence that circ_0020710 can directly bind to miR-370-3p in melanoma cells.

### Circ_0020710 upregulates CXCL12 level via sponging miR-370-3p

**Fig. 4:**
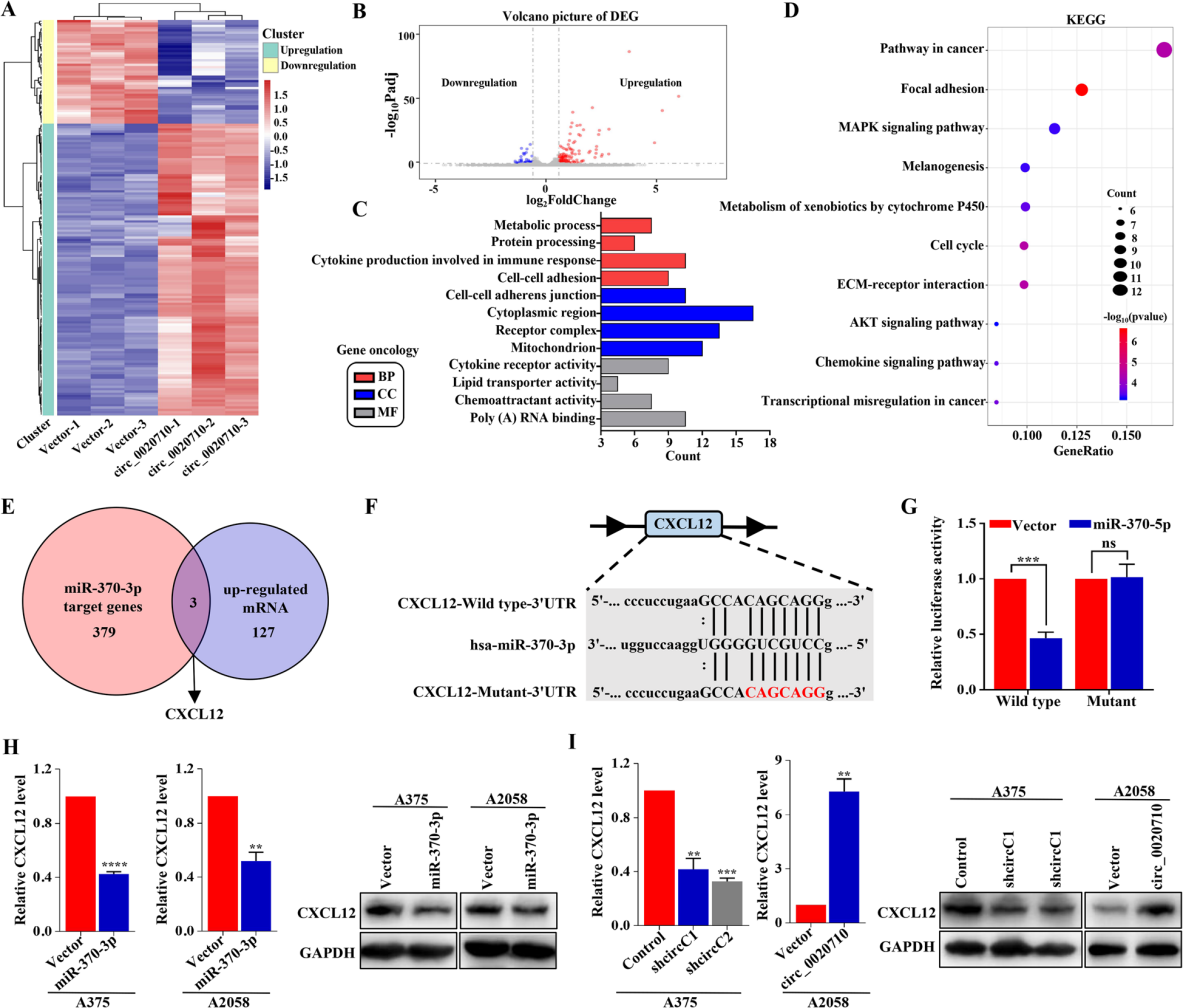
circ_0020710 upregulates the CXCL12 level via sponging miR-370-3p. **a** The heatmap of differentially expressed mRNAs in A2058 cells transfected with Vector or circ_0020710. Each sample was mixed with three replicates. **b** Volcano plot of differentially expressed mRNAs in A2058 cells transfected with Vector or circ_0020710. **c** and **d** GO and KEGG analyses of differentially expressed mRNAs in A2058 cells. **e** Schematic illustration exhibiting overlapping of miR-370-3p target mRNAs and upregulated DEGs. **f** Putative binding site of miR-370-3p with respect to CXCL12 was predicated via StarBase v3.0. **g** The luciferase activity of pLG3-CXCL12 in HEK-293 T cells after co-transfection with miR-370-3p. **h** and **i** Relative CXCL12 mRNA and protein expression in melanoma cells with different treatments. Unpaired student’s t-test and one-way ANOVA test were used for the statistical analyses. **p < 0.01; ***p < 0.001;****p < 0.0001; ns, no significant

According to previous studies, miR-370-3p could suppress certain oncogenes to inhibit tumor progression [20–22]. Thus, we hypothesized that circ_0020710 promoted tumor progression by protecting these oncogenes from downregulation by miR-370-3p. We carried out the transcriptomic analysis in melanoma cells, and found that 127 upregulated differently expressed genes (DEGs), and 45 downregulated DEGs in A2058-circ_0020710 cells compared with those in A2058-Vector cells (Fig. 4a and b). Gene Ontology (GO) analysis showed that these DEGs were mainly enriched in GO terms related to metabolic process, protein processing, cytokine production involved in immune response and cell-cell adhesion (biological process, BP); cell-cell adherens junction, cytoplasmic region, receptor complex and mitochondrion (cellular component, CC); cytokine receptor activity, lipid transporter activity, chemoattractant activity and poly (A) RNA binding (molecular function, MF) (Fig. 4c). Kyoto Encyclopedia of Genes and Genomes (KEGG) analysis were mainly enriched in pathway in cancer, MAPK signaling pathway, AKT signaling pathway and chemokine signaling pathway (Fig. 4d). Four databases (including miRanda, TargetScan, PITA and miRmap) were then employed to predict the potential targets of miR-370-3p, among which 379 mRNAs were obtained from the overlap (Fig. S4a). We then obtained the overlapped genes from the miR-370-3p targets and upregulated mRNAs, including CXCL12, KAT6A, C18orf25 (Fig. 4e). CXCL12 has been reported to promote tumor progression and predict poor prognosis in multiple tumors [23, 24]. Therefore, we speculated that circ_0020710 promoted the malignant progression of melanoma cells mainly through protecting CXCL12 from downregulation by miR-370-3p. We carried out a luciferase reporter assay transfecting with wild type or mutant CXCL12 sequence into HEK-293 T and A2058 cells. MiR-370-3p mimics significantly decreased the luciferase activity in cells containing the wild type CXCL12 sequence, but not the mutant CXCL12 sequence (Fig. 4f and g, Fig. S4b). Additionally, CXCL12 mRNA and protein levels were significantly decreased after miR-370-3p overexpression in A2058 and A375 cells (Fig. 4h). Moreover, CXCL12 mRNA and protein levels were also decreased after circ_0020710 downregulation in A375 cells, while being significantly increased after circ_0020710 upregulation in A2058 cells (Fig. 4i). Together, our results show that circ_0020710 upregulates CXCL12 expression via sponging miR-370-3p.

### Elevated CXCL12 promotes the proliferation, migration and invasion of melanoma cells

**Fig. 5:**
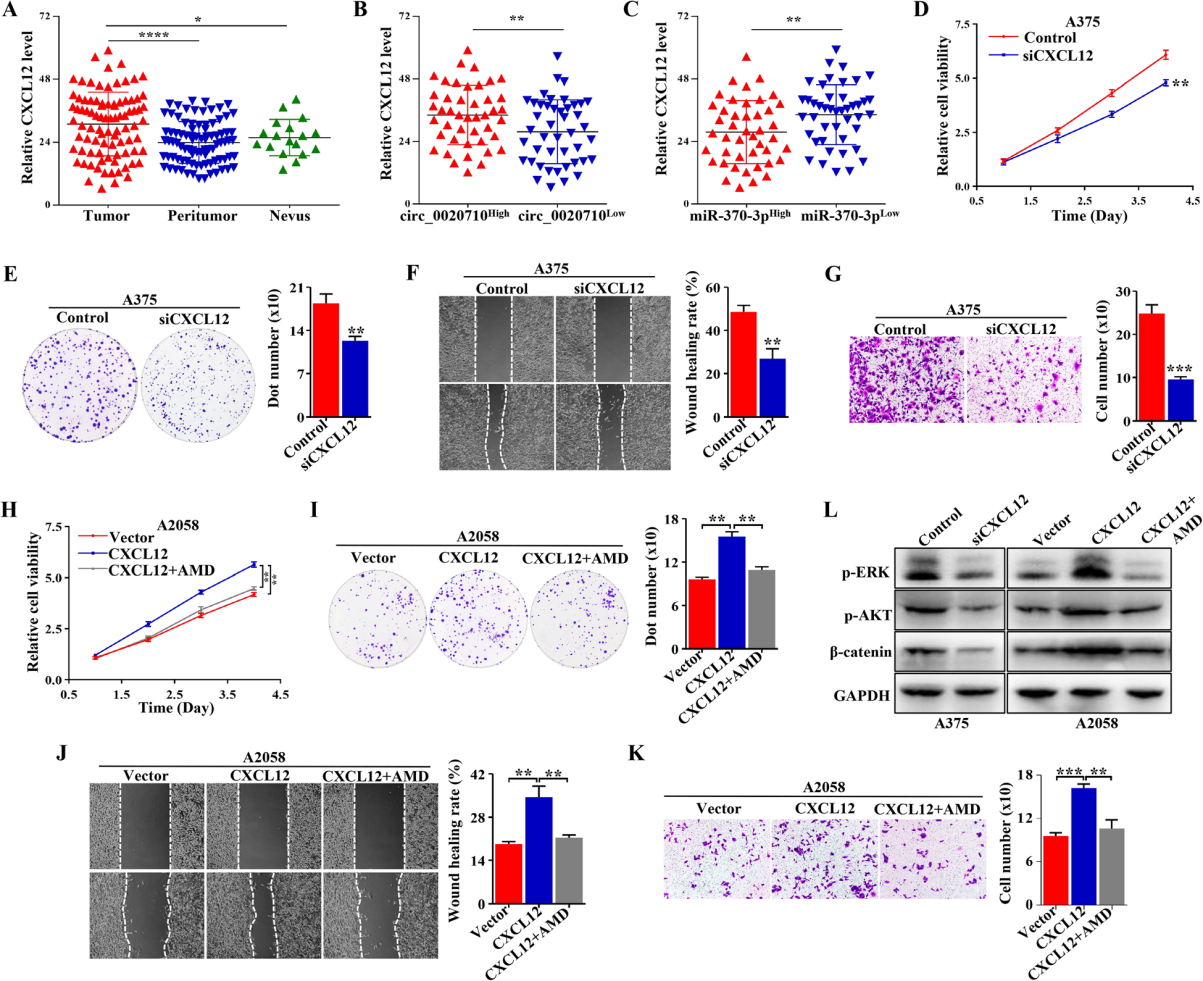
Elevated CXCL12 promotes melanoma progression. **a** IHC assay was used to detect the expression of CXLC12 in 88 paired melanoma and normal tissues and 18 benign nevi tissues. **b** Relative CXCL12 expression in different melanoma samples according to the expression of circ_0020710. **c** Relative CXCL12 expression in different melanoma samples according to the expression of miR-370-3p. **d-g** Colony formation, wound healing, transwell invasion, and CCK-8 assays performed in A375-Control and A375-siCXCL12 cells. **h-k** CCK-8, Colony formation, transwell invasion and wound healing assays performed in A2058 cells treated with Vector, CXCL12, and CXCL12 + AMD3100. **l** Western blot assay was used to detect the p-ERK, p-AKT, and β-catenin levels in melanoma cells with different treatment, GAPDH was used as a negative control. Paired student’s-t test, unpaired student’s t-test, Mann-Whitney U test, one-way ANOVA test and Kruskal-Wallis test were used for the statistical analyses. *p < 0.05; **p < 0.01; ***p < 0.001; ****p < 0.0001; ns, no significant

The potential role of CXCL12 was further explored in melanoma. Through IHC analysis, we presented that CXCL12 level was significantly upregulated in melanoma tissues compared with paired normal tissues and benign nevi tissues (Fig. 5a). Simultaneously, we found that CXCL12 level in the miR-370-3p^High^ group was lower than that in the miR-370-3p^Low^ group (Fig. 5b), and CXCL12 level in the circ_0020710^High^ group was higher than that in the circ_0020710^Low^ group (Fig. 5c). We then evaluated the roles of CXLC12 in melanoma by CXCL12 knockdown or overexpression. After the transfection efficiency was verified by western blot (Fig. S5a), CCK-8 and colony formation assays showed that the cell viability was inhibited after CXCL12 knockdown, and wound healing, transwell invasion assays revealed that CXLC12 knockdown decreased cell migration, invasion of melanoma cells (Fig. 5d-g). Adversely, CXCL12 overexpression promoted cell proliferation, migration, invasion, while these were dismissed by AMD3100, an inhibitor of the CXCL12/CXCR4 axis (Fig. 5h-k). Studies have reported that elevated CXCL12 activated multiple signals in tumor cells. We found that CXCL12 overexpression upregulated p-ERK, p-AKT, and β-catenin levels (Fig. 5l). These results indicate that upregulated CXLC12 promotes tumor progression and activates multiple pathways in melanoma.

### CXCL12 downregulation turnovers circ_0020710-induced malignant phenotype

**Fig. 6:**
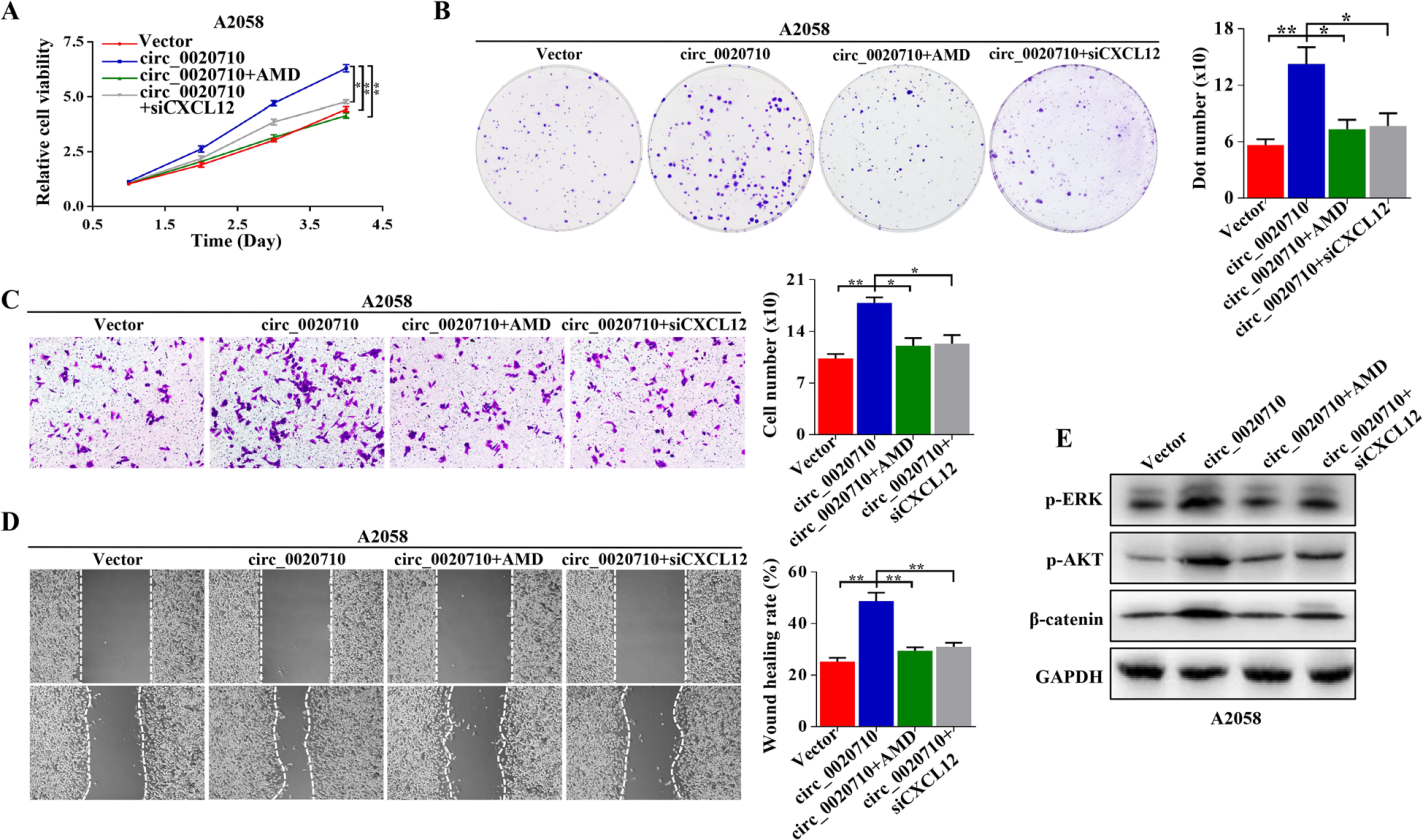
Downregulated CXCL12 turnovers circ_0020710-induced malignant phenotype. **a-d** CCK-8, Colony formation, transwell invasion and wound healing assays performed in A2058 cells treated with Vector, circ_0020710, circ_0020710 + AMD3100, and circ_0020710 + siCXCL12. **e** Western blot assay was used to detect the p-ERK, p-AKT, and β-catenin levels in melanoma cells following different treatments, GAPDH was used as a negative control. One-way ANOVA test and Kruskal-Wallis test were used for the statistical analyses. *p < 0.05; **p < 0.01

To further verify whether circ_0020710 induced melanoma progression through the miR-370-3p sponge, rescue experiments were carried out. A2058-circ_0020710 cells were treated with siCXCL12 or AMD3100, and then, CCK-8, colony formation, wound healing and transwell invasion assays were performed. The results showed that circ_0020710 overexpression induced proliferation, migration and invasion promotion were blocked by genetically or pharmacologically inhibiting the CXCL12/CXCR4 axis (Fig. 6a-d). In addition, circ_0020710 overexpression induced high p-ERK, p-AKT and β-catenin levels were also reduced by genetically or pharmacologically inhibiting the CXCL12/CXCR4 axis (Fig. 6e). These results show that CXCL12 downregulation reverses the circ_0020710-induced malignant phenotype of melanoma cells.

### circ_0020710 is correlated with cytotoxic lymphocyte exhaustion and anti-PD-1 therapy resistance

**Fig. 7:**
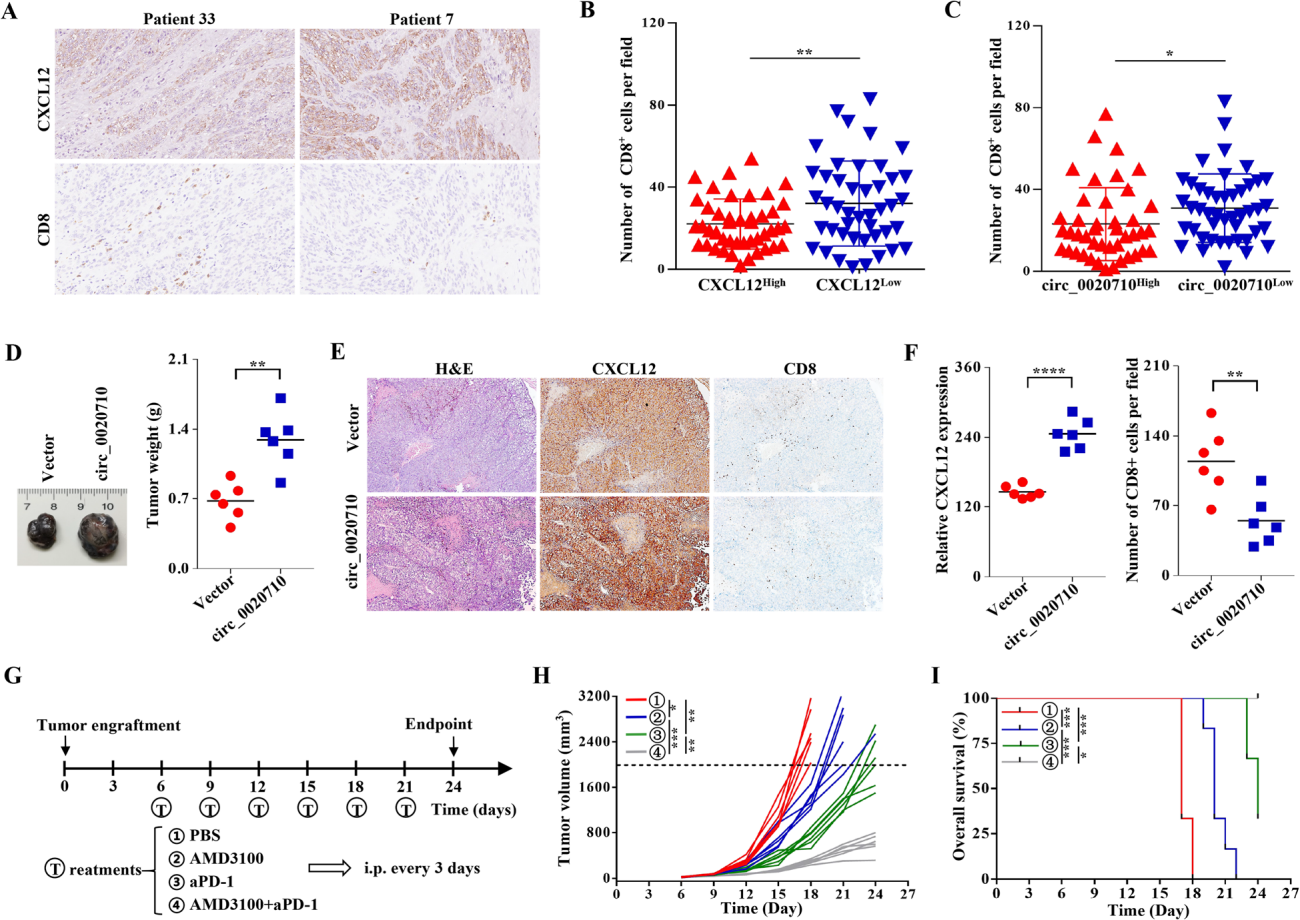
circ_0020710 is correlated with CTL exclusion. **a** IHC assay was used to detect the CTL number. **b** Number of CTL in different melanoma samples according to the expression of CXCL12. **c** Number of CTL in different melanoma samples according to the expression of circ_0020710. **e** Photographs illustrated tumors in xenografts. **f** IHC analysis was used to detect CTL numbers and CXLC12 expression. **g** Schematic representation of xenograft tumor model. **h** Growth curve of subcutaneous B16F10-circ_0020710 cells with different treatments. **i** Survival curve of part h. Unpaired student’s t-test, Mann-Whitney U test and one-way ANOVA test were used for the statistical analyses. *p < 0.05; **p < 0.01; ****p < 0.0001; ns, no significant

CXCL12 can recruit suppressive immune cells and eventually leads to the exhaustion of cytotoxic lymphocyte (CTL) [25, 26]. IHC assay was performed to explore the relationship between circ_0020710/CXCL12 expression and CTL infiltration. The infiltration number of CTL in samples with high CXCL12 and circ_0020710 expression was lesser than that in samples with low CXCL12 and circ_0020710 expression (Fig. 7a-c). Interestingly, human and mouse cells share the same miR-370-3p sequence, and the mouse Cxcl12 is also a speculated target of mmu-miR-370-3p (Fig. S6a). We then carried out a luciferase reporter assay using mmu-miR-370-3p mimics co-transfected with the luciferase reporter vectors (containing wild type or mutant mouse Cxcl12 sequence) into HEK-293 T cells. The luciferase activity was significantly attenuated by the mmu-miR-370-3p mimics in cells containing the wild type Cxcl12 sequence, but not in those containing the mutant Cxcl12 sequence (Fig. S6b). Furthermore, our results showed that Cxcl12 was significantly upregulated when circ_0020710 was overexpressed in B16F10 cells (Fig. S6c). To explore the effects of circ_0020710 on CTL infiltration, we established a subcutaneous xenograft tumor model using C57BL/6 mice. We found that the tumors in the B16F10-circ_0020710 group were significantly heavier than those in the B16F10-Vector group (Fig. 7d). Through IHC analysis, we confirmed that CXCL12 expression was upregulated in the B16F10-circ_0020710 group compared with that in the B16F10-Vector group (Fig. 7e and f). Meanwhile, we also found that high level of circ_0020710 was accompanied by a low extent of CTL infiltration, which indicated that upregulation of the circ_0020710/miR-370-3p/CXCL12 axis induced an immunosuppression microenvironment (Fig. 7e and f). A majority of cancer patients fail to respond to anti-PD-(L)1 therapy due to multiple immunosuppressive mechanisms including lack of CTL infiltration [27]. We proposed that the inhibition of the circ_0020710/CXCL12 axis might promote the effects of anti-PD-1 therapy. We then evaluated the anti-tumor effects of AMD3100 and anti-PD-1 combination using the subcutaneous xenograft tumor model. Once tumors were accessible, mice were treated with PBS, anti-PD-1, AMD3100, or the combination of anti-PD-1 and AMD3100 until the study endpoint (Fig. 7g). Meanwhile, no significant changes in average mice body weight or toxicity in the liver and kidney were observed (data not shown). This combination significantly inhibited tumor growth compared with the control treatment or treatment with single agent (Fig. 7h), and the mice in the combination group showed the best prognosis (Fig. 7i). Overall, the above results indicate that the inhibiter of circ_0020710/CXCL12 axis promotes the therapeutic efficacy of anti-PD-1 treatment.

## Discussion

Through advanced sequencing and bioinformatics methods, a large proportion of the transcriptome is discovered to be spliced into single-stranded covalently closed circular transcripts. Studies have shown that circRNAs are much more stable and abundant compared with linear RNA, and present a tissue-specific expression pattern [5]. Unfortunately, although the biological function of circRNAs has attracted much attention, few studies have revealed its roles in melanoma. Here, we speculated that oncoprotein CD151 derived circRNAs might have certain roles in tumor promotion. We detected 18 circRNAs derived from CD151, and among them, circ_0020710 was generally overexpressed in all of the melanoma tissues compared with paired normal tissues. Through our clinical samples, we confirmed that circ_0020710 was significantly upregulated in melanoma tissues and high level of circ_0020710 was positively correlated with poor prognosis. Through a series in vitro and in vivo experiments, we further identified that circ_0020710 could induce melanoma progression and immune escape, which strongly indicated its roles in melanoma promotion. Our research will undoubtedly enrich the research field of non-coding RNA in melanoma.

Studies have shown that circRNA participates in multiple regulatory mechanisms, such as ceRNAs, protein interactions, and gene transcriptional and translational regulation [28]. The ceRNA hypothesis indicates that circRNA serves as a miRNA sponge to eliminate miRNA inhibition on its target genes [29]. For example, circular RNA circAKT3, harboring multiple conserved binding sites for miRNA and is highly expressed in cisplatin-resistance gastric cancer samples, was recently reported to function as a miR-198 sponge to regulate PIK3R1 expression recently [30]. In another example, circTADA2A promotes osteosarcoma progression and metastasis, and regulates the CREB3 level by sponging miR-203a-3p [31]. In the present study, we showed that circ_0020710 is mainly located in the cytoplasm of melanoma cells, and the RIP assay further confirmed that circ_0020710 acts as a sponge for miR-370-3p (studies have confirmed that miR-370-3p is a tumor suppressor) without influencing miR-370-3p expression. MiRNA is an important post-transcriptional regulator, resulting in a decrease in mRNA stability through direct base pairing with mRNA 3′-UTR target sites [32]. Combining with the results of miRNA target prediction and RNA-seq, we obtained three potential miR-370-3p target genes, including CXCL12, KAT6A, and C18orf25. CXCL12 is a well-known oncogene in multiple tumors and was selected for the following research. All these findings above indicate that circ_0020710 serves as a ceRNA to contribute to melanoma progression through the miR370-3p/CXCL12 axis.

CXCL12, the most studied chemokine family member, is a homeostatic chemokine highly produced in multiple tissues and cells. CXCL12 exerts its functions by interacting with CXC chemokine receptor 4 (CXCR4) and atypical chemokine receptor 3 (ACKR3, also known as CXCR7) through autocrine, paracrine and other manners [33, 34]. The CXCL12/CXCR4/CXCR7 axis plays key roles in many physiological and pathological processes, including embryogenesis, wound healing, angiogenesis, tumor development, as well as recruiting suppressive immune cells. For example, CXCL12 is generally overexpressed in adamantinomatous craniopharyngiomas, and the CXCL12/CXCR4 axis promotes tumor proliferation, migration, and invasion through PI3K/AKT signal pathway [35]. In another example, treatment with AMD3100, a selective CXCR4 antagonist, resulted in increased tumor cell apoptosis and necrosis, and selective reduction of foxp3^+^ regulatory T cells and inducing an immunosuppressive microenvironment [36]. In the present study, we showed that circ_0020710 serves as a miR-370-3p sponge to decrease CXCL12 inhibition, resulting in the activation of CXCL12/CXCR4/CXCR7 axis. On the one hand, elevated CXCL12 levels promoted melanoma progression through autocrine and paracrine pathways; on the other hand, elevated CXCL12 levels promoted immune suppressor cells recruitment to induce an immunosuppressive microenvironment, resulting in reduced CTL infiltration. Since the effect of anti-PD(L)1 therapy mainly depends on the number and activity of CTLs, we evaluated the anti-tumor effects of the combination of AMD3100 and anti-PD-1, and found it significantly inhibited tumor growth, compared to the case for the control treatment or treatment with each agent alone.

## Conclusion

**Fig. 8:**
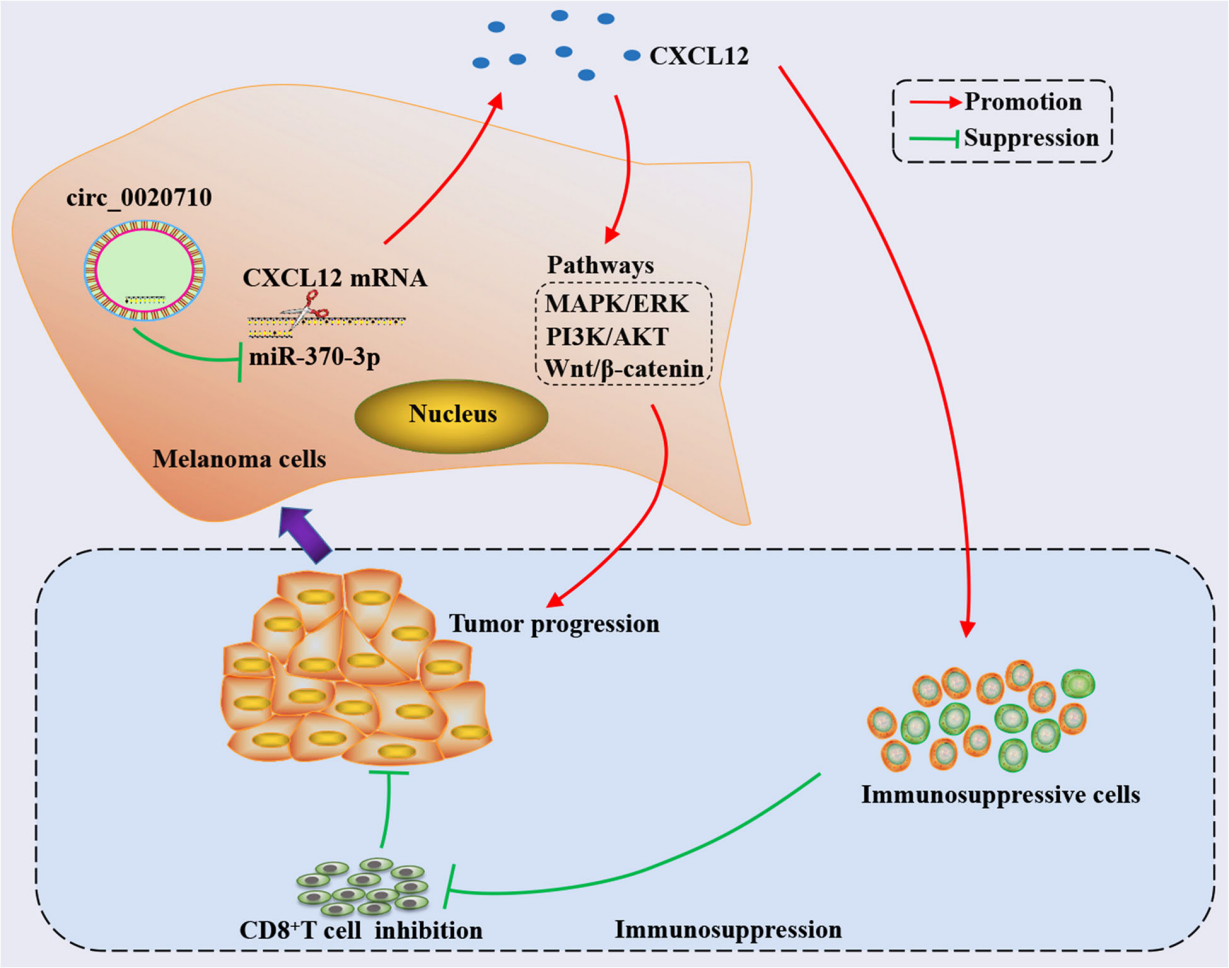
A schematic diagram depicting the biological function and mechanism of circ_0020710 in melanoma

In summary, we detected a novel circRNA (circ_0020710, derived from CD151) that was overexpressed in melanoma tissues, and high level of circ_0020710 positively was correlated with the poor prognosis of melanoma patients. Our results not only elucidate the potential mechanism by which circRNAs regulate the malignant progression and immune escape of melanoma, but also suggest that the circ_0020710/ CXCL12 axis could be a potential therapeutic target for melanoma patients (Fig. 8).

## Supplementary information

**Additional file 1: Fig. S1** Expression of CD151 and circ_0020710 after RNase R treatment. **a** and **b** qRT-PCR analyses of CD151 and circ_0020710 RNA level after treatment with RNase R in A375 and Sk-mel-28 cells.

**Additional file 2: Fig. S2** Relative circ_0020710 and CD151 expression. **a** qRT-PCR analysis of circ_0020710 level in different melanoma cell lines and HaCaT, a normal epidermal cell line. **b** qRT-PCR analysis of CD151 expression after circ_0020710 interference and over-expression. **c** Western blot assay was used to detect the PCNA, CDK1, CDK2 levels in melanoma cells following different treatments, GAPDH was used as a negative control. Unpaired student’s t-test and one-way ANOVA test were used for the statistical analyses. ns, no significant.

**Additional file 3: Fig. S3** circ_0020710 acted as a sponge of miR-370-3p without regulating its expression. **a** and **b** qRT-PCR analysis of circ_0020710, GAPDH, and U6 levels in the cytoplasm and nucleus in A375 and A2058 melanoma cells. **c** RIP assay for circ_0020710 level in HEK-293 cell. **d** The luciferase activity of pLG3-circ_0020710 in A2058 cells after co-transfection with miR-370-3p. **e** and **f** Relative miR-370-3p and circ_0020710 expression in melanoma cells with different treatments analyzed by qRT-PCR. Unpaired student’s t-test and one-way ANOVA test were used for the statistical analyses. **p < 0.01; ns, no significant.

**Additional file 4: Fig. S4** CXCL12 is a target of miR-370-3p. **a** Schematic illustration exhibiting the overlapping of the target mRNAs of miR-370-3p predicted by miRanda, PITA, TargetScan, and miRmap database. **b** The luciferase activity of pLG3-circ_0020710 in A2058 cells after co-transfection with miR-370-3p. Unpaired student’s t-test was used for the statistical analyses. ***p < 0.001; ns, no significant.

**Additional file 5: Fig. S5** Western blot assay was used to detect the expression of CXCL12, GAPDH was used as a negative control.

**Additional file 6: Fig. S6** Cxcl12 is a target of mmu-miR-370-3p in mouse B16F10 cells. **a** Putative binding site of mmu-miR-370-3p with respect to Cxcl12 was predicated via StarBase v3.0. **b** The luciferase activity of pLG3-Cxcl12 in HEK-293T cells after co-transfection with mmu-miR-370-3p. **c** Relative Cxcl12 expression in melanoma cells following different treatments. Unpaired student’s t-test was used for the statistical analyses. **p<0.01; ns, no significant.

**Additional file 7: Table S1** Sequences of Primers used for qRT-PCR in this study. **Table S2** List of Primary Antibodies Used in this Study. **Table S3**. Target sequences of circ_0020710 shRNAs. **Table S4** circ_0020710 circRIP probe sequence.

**Additional file 8.** Supplementary Materials and Methods.

## Data Availability

All data generated or analyzed during this study are included either in this article or in the supplementary Materials and Methods, Tables, Figures, and Figure Legends files.

## Abbreviations

circRNAs: Circular RNAs
qRT-PCR: Quantitative real-time polymerase chain reaction
OS: Overall survival
ncRNAs: Non-coding RNAs
EMT: Epithelial-mesenchymal transition
shRNAs: Small hairpin RNAs
cDNA: Complementary DNA
siRNA: Small interfering RNA
IHC: Immunohistochemistry
FISH: Fluorescence in situ hybridization
SD: Standard deviation
AUC: Area under the curve
RIP: RNA immunoprecipitation assay
DEGs: Differently expressed genes
GO: Gene Ontology
BP: Biological process
CC: Cellular component
MF: Molecular function
KEGG: Kyoto Encyclopedia of Genes and Genomes
CTL: Cytotoxic lymphocyte
CXCR4: CXC chemokine receptor 4
