# Supplementary figures and images for: Circular RNA circ_0020710 drives tumor progression and immune evasion by regulating the miR-370-3p/CXCL12 axis in melanoma
Source: Mol Cancer. 2020 May 7;19:84. doi: 10.1186/s12943-020-01191-9 (PMC7204052; doi:10.1186/s12943-020-01191-9)

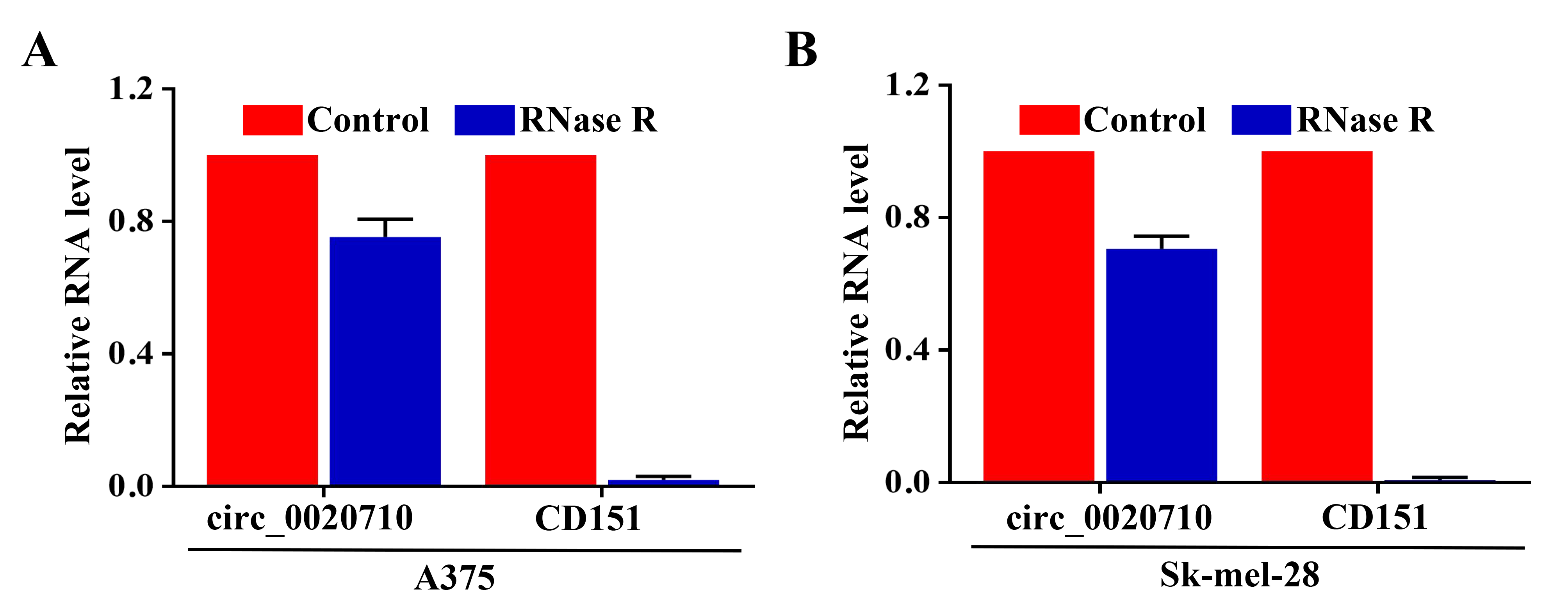

Supplement: Supplementary file 1 — Additional file 1: Fig. S1 Expression of CD151 and circ_0020710 after RNase R treatment. a and b qRT-PCR analyses of CD151 and circ_0020710 RNA level after treatment with RNase R in A375 and Sk-mel-28 cells. [file 12943_2020_1191_MOESM1_ESM.tif]

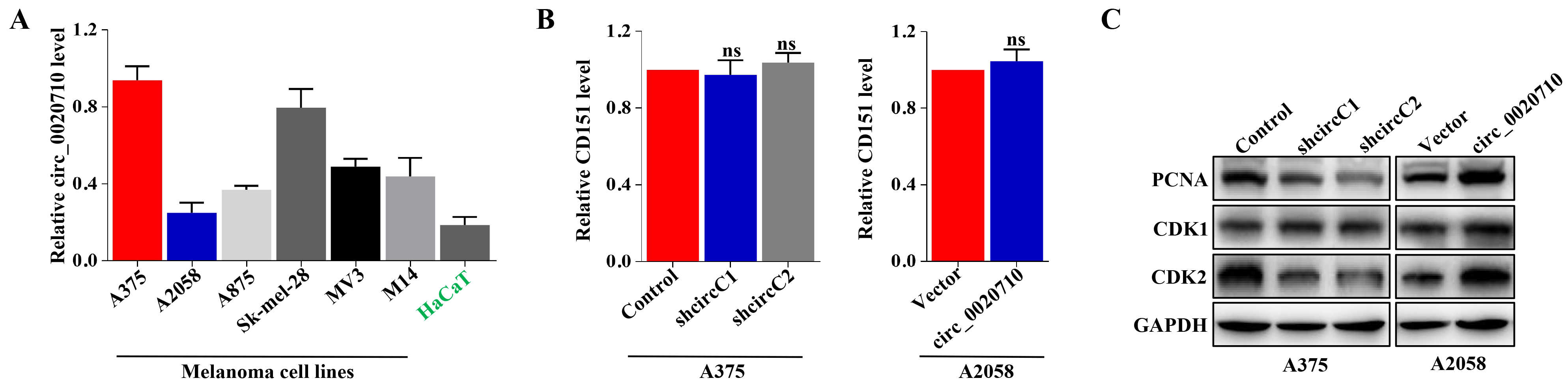

Supplement: Supplementary file 2 — Additional file 2: Fig. S2 Relative circ_0020710 and CD151 expression. a qRT-PCR analysis of circ_0020710 level in different melanoma cell lines and HaCaT, a normal epidermal cell line. b qRT-PCR analysis of CD151 expression after circ_0020710 interference and over-expression. c Western blot assay was used to detect the PCNA, CDK1, CDK2 levels in melanoma cells following different treatments, GAPDH was used as a negative control. Unpaired student’s t-test and one-way ANOVA test were used for the statistical analyses. ns, no significant. [file 12943_2020_1191_MOESM2_ESM.tif]

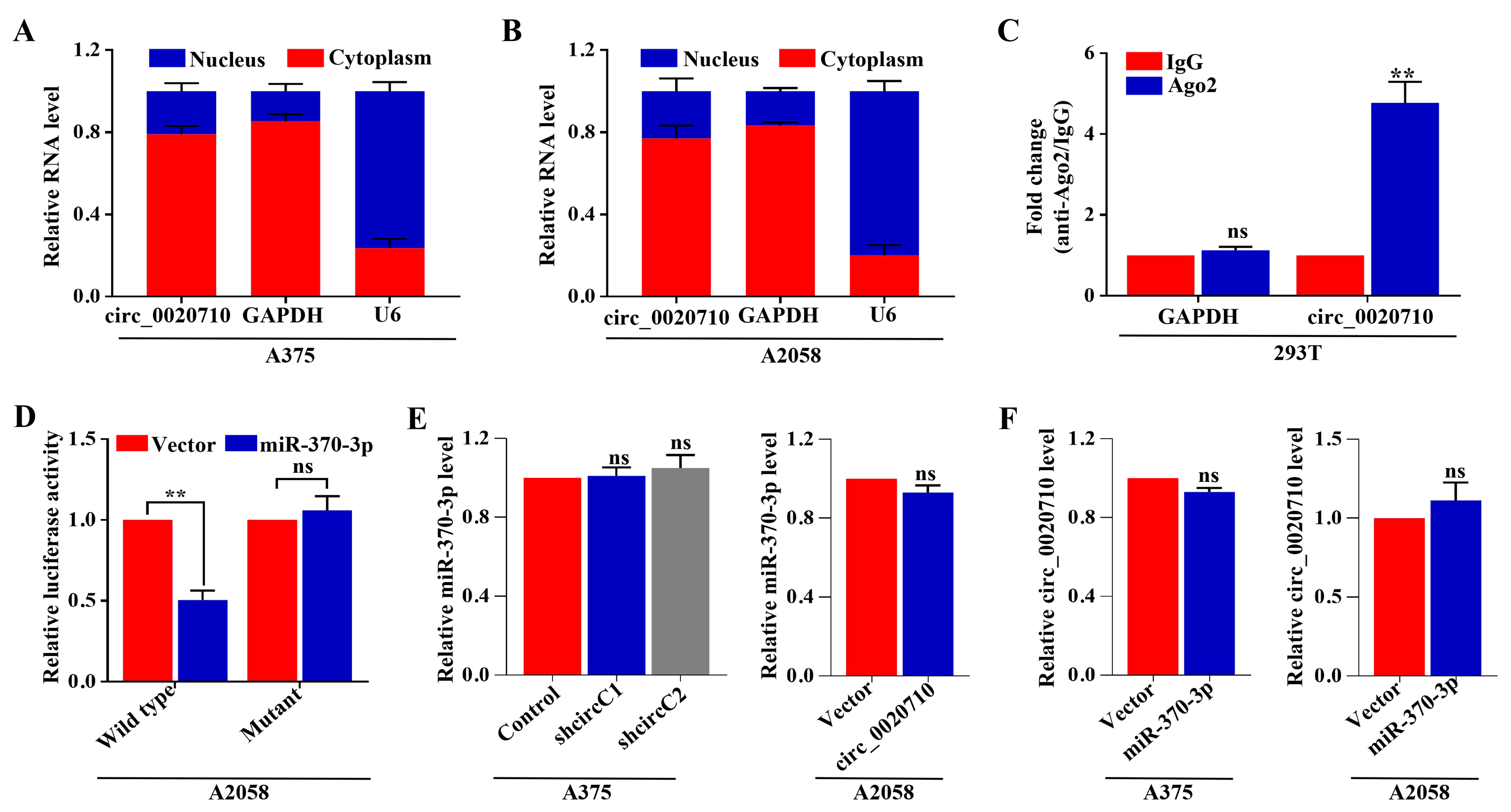

Supplement: Supplementary file 3 — Additional file 3: Fig. S3 circ_0020710 acted as a sponge of miR-370-3p without regulating its expression. a and b qRT-PCR analysis of circ_0020710, GAPDH, and U6 levels in the cytoplasm and nucleus in A375 and A2058 melanoma cells. c RIP assay for circ_0020710 level in HEK-293 cell. d The luciferase activity of pLG3-circ_0020710 in A2058 cells after co-transfection with miR-370-3p. e and f Relative miR-370-3p and circ_0020710 expression in melanoma cells with different treatments analyzed by qRT-PCR. Unpaired student’s t-test and one-way ANOVA test were used for the statistical analyses. **p < 0.01; ns, no significant. [file 12943_2020_1191_MOESM3_ESM.tif]

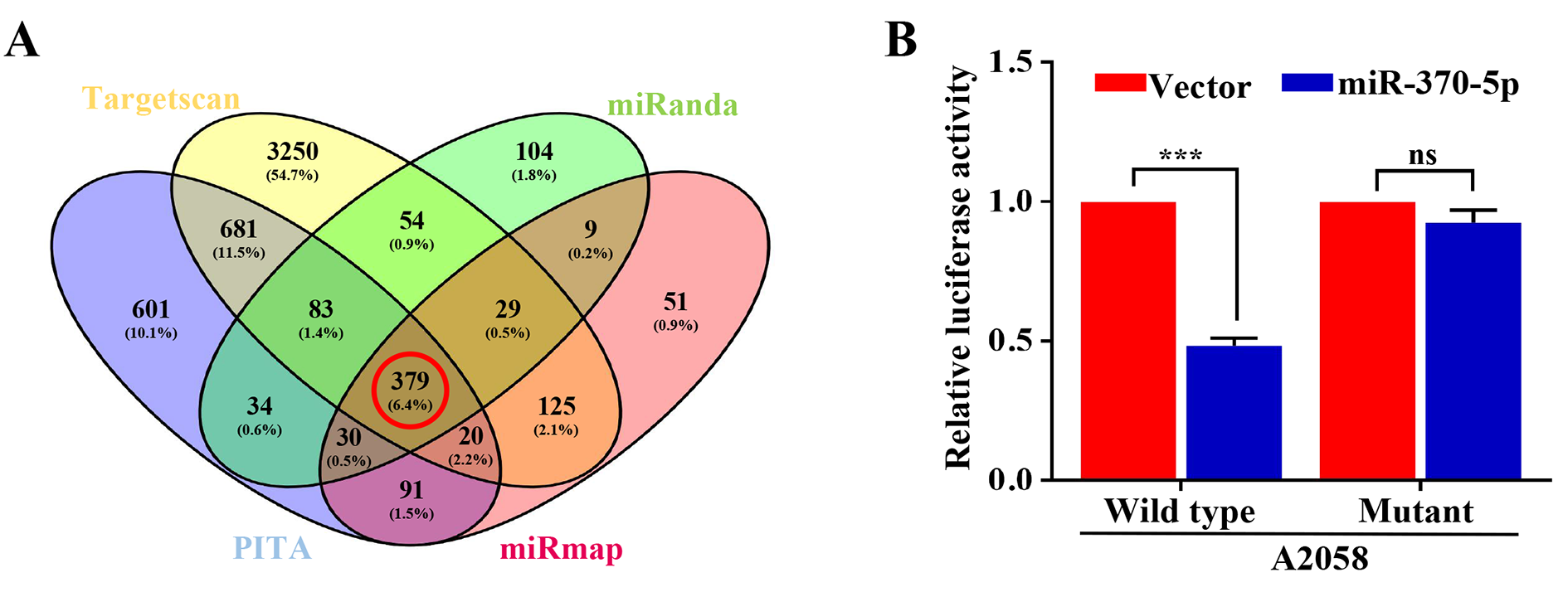

Supplement: Supplementary file 4 — Additional file 4: Fig. S4 CXCL12 is a target of miR-370-3p. a Schematic illustration exhibiting the overlapping of the target mRNAs of miR-370-3p predicted by miRanda, PITA, TargetScan, and miRmap database. b The luciferase activity of pLG3-circ_0020710 in A2058 cells after co-transfection with miR-370-3p. Unpaired student’s t-test was used for the statistical analyses. ***p < 0.001; ns, no significant. [file 12943_2020_1191_MOESM4_ESM.tif]

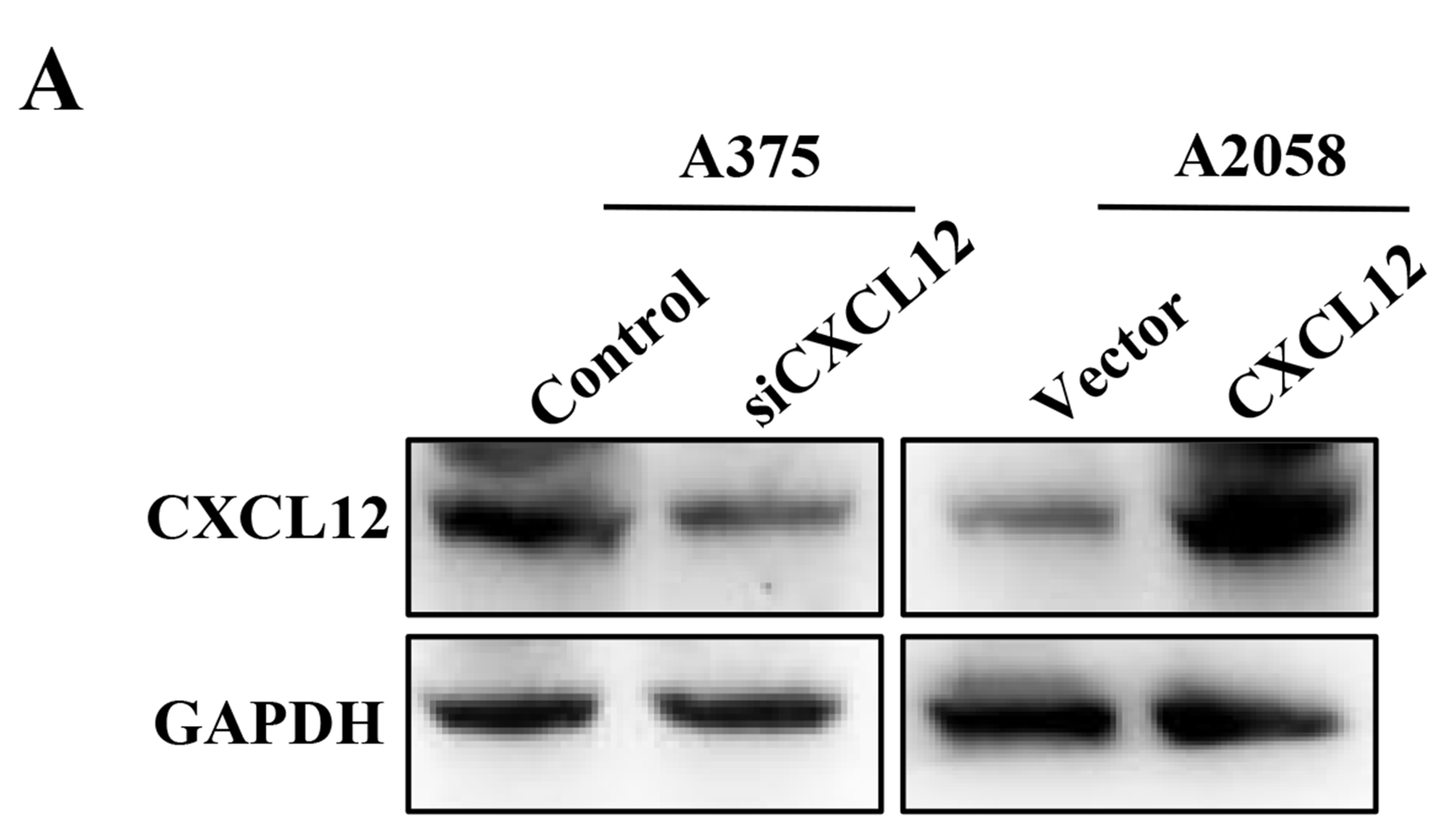

Supplement: Supplementary file 5 — Additional file 5: Fig. S5 Western blot assay was used to detect the expression of CXCL12, GAPDH was used as a negative control. [file 12943_2020_1191_MOESM5_ESM.tif]

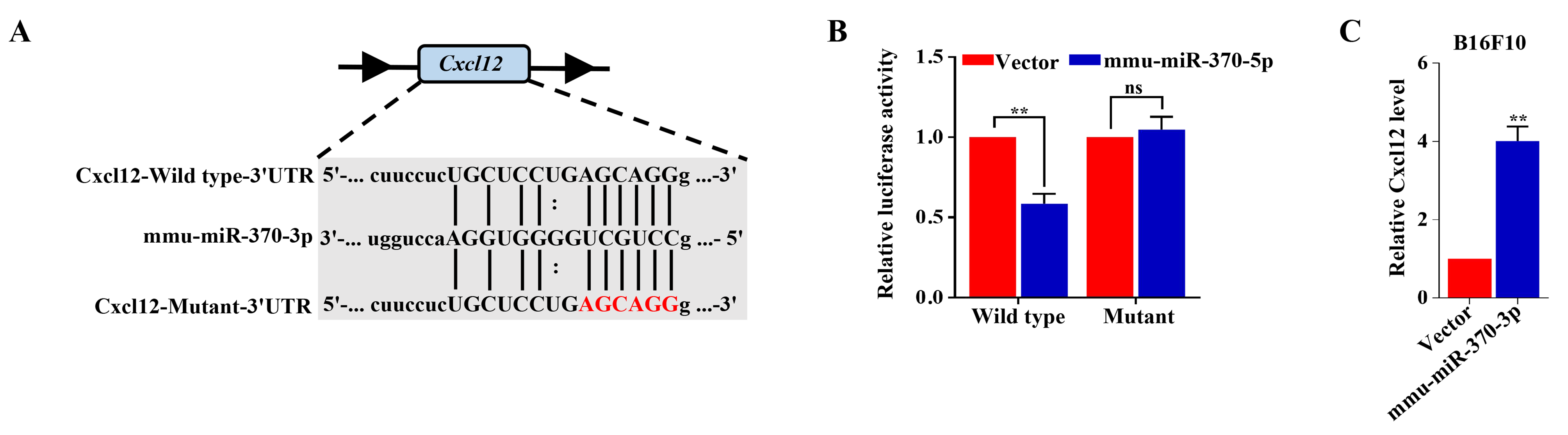

Supplement: Supplementary file 6 — Additional file 6: Fig. S6 Cxcl12 is a target of mmu-miR-370-3p in mouse B16F10 cells. a Putative binding site of mmu-miR-370-3p with respect to Cxcl12 was predicated via StarBase v3.0. b The luciferase activity of pLG3-Cxcl12 in HEK-293T cells after co-transfection with mmu-miR-370-3p. c Relative Cxcl12 expression in melanoma cells following different treatments. Unpaired student’s t-test was used for the statistical analyses. **p<0.01; ns, no significant. [file 12943_2020_1191_MOESM6_ESM.tif]
