# Supplementary material for: Circular RNA circ_0020710 drives tumor progression and immune evasion by regulating the miR-370-3p/CXCL12 axis in melanoma
Source: Mol Cancer. 2020 May 7;19:84. doi: 10.1186/s12943-020-01191-9 (PMC7204052; doi:10.1186/s12943-020-01191-9)
Supplement: Supplementary file 7 — Additional file 7: Table S1 Sequences of Primers used for qRT-PCR in this study. Table S2 List of Primary Antibodies Used in this Study. Table S3. Target sequences of circ_0020710 shRNAs. Table S4 circ_0020710 circRIP probe sequence. [file 12943_2020_1191_MOESM7_ESM.zip › Table S1.docx]

**Table S1 Sequences of Primers used for qRT-PCR.**

| **hsa_circ_0020712** |  |
| --- | --- |
| Forward | 5’-ACAATTGCTGCTTCTGGCCTA-3’ |
| Reverse | 5’-GCAGCAATTGTAGGTAAACAG-3’ |
| **hsa_circ_0020717** |  |
| Forward | 5’-ACGCCTACTACCAGCAGCTGG-3’ |
| Reverse | 5’-CCAGTCACCATGACGACAGTG-3’ |
| **hsa_circ_0020727** |  |
| Forward | 5’-ACACTTGTAGAGCAGAATGC-3’ |
| Reverse | 5’-CCAAGGGTCAGAGCTCACCTG-3’ |
| **hsa_circ_0020722** |  |
| Forward | 5’-CTTGTAGAGCAGATGGATTCC-3’ |
| Reverse | 5’-CCTCCACCTTGTAGATGTTGG-3’ |
| **hsa_circ_0020719** |  |
| Forward | 5’-ACTTGTAGAGCAGATGGATAC-3’ |
| Reverse | 5’-ACAGCAGTGGAACTCCTGCTG-3’ |
| **hsa_circ_0020724** |  |
| Forward | 5’-CTTGTAGAGCAGATGGAGGCG-3’ |
| Reverse | 5’-TGCTCCAGCTTGAGACTCCTG-3’ |
| **hsa_circ_0020725** |  |
| Forward | 5’-AACACTTGTAGAGCAGAGTCT-3’ |
| Reverse | 5’-GAGGTTATGGTGATGGCACAG-3’ |
| **hsa_circ_0020714** |  |
| Forward | 5’-TCGGAACCTGCTGCGCCTGCC-3’ |
| Reverse | 5’-AGGCTGATGTAGTCACTCTTG-3’ |
| **hsa_circ_0020713** |  |
| Forward | 5’-AACACTTGTAGAGCAGACCTA-3’ |
| Reverse | 5’-GCCAGCCAGAAGCAGCAATTG-3’ |
| **hsa_circ_0020723** |  |
| Forward | 5’-AACACTTGTAGAGCAGAGGCG-3’ |
| Reverse | 5’-CCCAGAGGTTATGGTGATGGC-3’ |
| **hsa_circ_0020710** |  |
| Forward | 5’-CACTTGTAGAGCAGAATTCTC-3’ |
| Reverse | 5’-CGTTGAACTCACCCATCCTGG-3’ |
| **hsa_circ_0020711** |  |
| Forward | 5’-CTTGTAGAGCAGATGGAATTC-3’ |
| Reverse | 5’-TCGTTGAACTCACCCATCCTG-3’ |
| **hsa_circ_0020718** |  |
| Forward | 5’-GCATTGCCTGTGTGCAGTACT-3’ |
| Reverse | 5’-CGCTTGGTCATGGTGTCCTTC-3’ |
| **hsa_circ_0020720** |  |
| Forward | 5’-AACACTTGTAGAGCAGACTGA-3’ |
| Reverse | 5’-TCTCGCCAGTCCTGTGAGTTG-3’ |
| **hsa_circ_0020726** |  |
| Forward | 5’-CTTGTAGAGCAGATGGAGTCT-3’ |
| Reverse | 5’-GAGGTTATGGTGATGGCACAG-3’ |
| **hsa_circ_0020716** |  |
| Forward | 5’-ACTTGTAGAGCAGATGGACCC-3’ |
| Reverse | 5’-GCTGATGTAGTCACTCTTGAG-3’ |
| **hsa_circ_0020721** |  |
| Forward | 5’-CTTGTAGAGCAGATGGACTGA-3’ |
| Reverse | 5’-ATCCACTCACTGTCTCGCCAG-3’ |
| **hsa_circ_0020715** |  |
| Forward | 5’-GGACCAGCTGCAGCAGGAGCC-3’ |
| Reverse | 5’-AGGCTGATGTAGTCACTCTTG-3’ |
| **CD151** |  |
| Forward | 5’-ATGGGTGAGTTCAACGAGAAGA-3’ |
| Reverse | 5’-GCAGGCTGATGTAGTCACTCT-3’ |
| **circANRIL** |  |
| Forward | 5’-AGAGAGAATTTTGACAGTGTC-3’ |
| Reverse | 5’-CCAGCACACCTAACAGTGATG-3’ |
| **GAPDH** |  |
| Forward | 5’-GGTATGACAACGAATTTGGC-3’ |
| Reverse | 5’-GAGCACAGGGTACTTTATTG-3’ |
| **CXCL12** |  |
| Forward | 5’-TTCCATTTGCAAGGGAAAAG-3’ |
| Reverse | 5’-ACACACAGCCAGTCAACGAG-3’ |
