# Supplementary material for: Circular RNA circ_0020710 drives tumor progression and immune evasion by regulating the miR-370-3p/CXCL12 axis in melanoma
Source: Mol Cancer. 2020 May 7;19:84. doi: 10.1186/s12943-020-01191-9 (PMC7204052; doi:10.1186/s12943-020-01191-9)
Supplement: Supplementary file 7 — Additional file 7: Table S1 Sequences of Primers used for qRT-PCR in this study. Table S2 List of Primary Antibodies Used in this Study. Table S3. Target sequences of circ_0020710 shRNAs. Table S4 circ_0020710 circRIP probe sequence. [file 12943_2020_1191_MOESM7_ESM.zip › Table S2.docx]

**Table S2 List of Primary Antibodies Used in the Study.**

| Antibody | Applications | Company |
| --- | --- | --- |
| CXCL12 | WB, IHC, IF, ELISA | Proteintech (17402-1-AP) |
| GAPDH | WB, IF, ELISA | Abcam (ab8245) |
| p-ERK | WB, IF, IHC, IP | CST (4370) |
| β-catenin | WB, F, IF, IHC, IP, IHC | CST (8480) |
| p-AKT | WB, IP, IF, IHC | CST (4060) |
| CD8 | WB, IHC, IF | Abcam (ab93278) |
| CD8 | IHC, IF, F | Abcam (ab22378) |
| AGO2 | WB, IHC, IP, CHIP, RIP | Abcam (ab32381) |
| IgG | WB, IHC, IP, CHIP, F | Abcam (ab172730) |

**Abbreviations:** WB, western blot; IHC, immunohistochemistry; IF, immunofluorescence; IP, immunoprecipitation; ELISA, enzyme-linked immunosorbent assay; F, flow cytometric analysis; RIP, RNA immunoprecipitation; CHIP, Chromatin Immunoprecipitation.
