# Supplementary material for: Circular RNA circ_0020710 drives tumor progression and immune evasion by regulating the miR-370-3p/CXCL12 axis in melanoma
Source: Mol Cancer. 2020 May 7;19:84. doi: 10.1186/s12943-020-01191-9 (PMC7204052; doi:10.1186/s12943-020-01191-9)
Supplement: Supplementary file 7 — Additional file 7: Table S1 Sequences of Primers used for qRT-PCR in this study. Table S2 List of Primary Antibodies Used in this Study. Table S3. Target sequences of circ_0020710 shRNAs. Table S4 circ_0020710 circRIP probe sequence. [file 12943_2020_1191_MOESM7_ESM.zip › Table S4 .docx]

**Table S4. circ_0020710 circRIP probe sequence.**

| Name | Sequence |
| --- | --- |
| circ_0020710 circRIP probe | CTCCCAGCGCTGAGAATTCTGCTCTACAAGTGTTTATTGAGCACCTGTATGTGGCAGGGATG |
