# Supplementary material for: Circular RNA circ_0020710 drives tumor progression and immune evasion by regulating the miR-370-3p/CXCL12 axis in melanoma
Source: Mol Cancer. 2020 May 7;19:84. doi: 10.1186/s12943-020-01191-9 (PMC7204052; doi:10.1186/s12943-020-01191-9)
Supplement: Supplementary file 8 — Additional file 8. Supplementary Materials and Methods. [file 12943_2020_1191_MOESM8_ESM.doc]

**Supplementary Materials and Methods**

**QRT-PCT and western blot assays**

Total RNA was extracted using TRIzol reagent (Invitrogen, Carlsbad, CA, USA) and reverse-transcribed using the cDNA Synthesis kit (Takara, Japan). The RNA quantity and density were verified by a spectrophotometer. QRT-PCR was performed using the SYBR Green Master MIX Kit (Takara, Japan) according to the manufacturer’s instructions. The assays were operated in triplicate and relative gene expression was determined by using the 2^-ΔΔCt^ method [16].

For total protein extraction, cell lysates were obtained using RIPA buffer (Beyotime, Shanghai, China) supplemented with phosphatase and protease inhibitors (Yeasen, Shanghai, China). A total of 15 μL protein was injected into a Bis-Tris SDS/PAGE gel and transferred to PVDF membranes. After blocking with 5% BSA, the membranes were incubated with primary antibody overnight at 4°C. Membranes were then exposed to the secondary antibody for 60 min. Bands were incubated with an ECL kit and analyzed with an imaging system. Densitometric analysis was carried out using Adobe Photoshop CS6.

**Immunohistochemistry assay**

Slides were deparaffinized and rehydrated following the manufacturer’s instructions. After incubation in 0.3% H_2_O_2_, antigen retrieval was conducted in citrate buffer. Then, they were incubated with primary antibodies overnight at 4 °C, and then with secondary antibodies for 1 hour. The sections were then incubated with a DAB kit (Gene Tech, Shanghai, China). Images were obtained from each area using a standard Olympus Microscope (Olympus, Japan). For IHC quantification, the slides were viewed independently by two pathologists who were unaware of the patients’ clinical information, and disagreements were resolved by reaching a consensus. The score was determined based on a combination of staining percentage and intensity as described previously [15].

**CCK-8, colony formation, wound-healing migration, and Matrigel invasion assays**

For CCK-8 assay, 1000 cells were inoculated into each well of 96-well plates. At each time point (24, 48, 72 and 96 h), 10 μL CCK-8 solution (Yeasen, Shanghai, China) was added into the sextuplicate wells. The wells were incubated for 3 h, and the absorbance of each individual well was determined at 490 nm. Obtained data are presented as a line chart.

For colony formation assay, cells were digested into single-cell suspension and 1000 cells seeded in each culture dish (6 cm). The appropriate complete medium was added to each dish with the culture medium being refreshed every three days. The cells were then washed with PBS, fixed with 4% paraformaldehyde and stained with 0.4% crystal violet for 15 min. The number of colonies containing >10 cells was counted manually and averaged from the duplicate wells.

For wound-healing migration assay, the cell monolayers were mechanically disrupted using a sterile 200 μl micropipette tip to generate a linear wound. The average distance migrated by the cells was measured using a microscope calibrated with an ocular micrometer.

For Matrigel invasion assays, cells were incubated using 24-well transwell plates (8 μm pore size, Corning, NY, USA). One million cells suspended in serum-free medium were plated in the upper chambers with Matrigel (BD Biosciences, USA), and 0.6 ml medium with 10% FBS was added to the lower chamber. After incubation for a suitable amount of time, the cells were fixed in 4% paraformaldehyde, stained by crystal violet and counted under a microscope.
